# Wealth and antenatal care use: implications for maternal health care utilisation in Ghana

**DOI:** 10.1186/2191-1991-2-14

**Published:** 2012-08-06

**Authors:** Eric Arthur

**Affiliations:** 1 Department of Economics and Statistics, University of Benin, Nigeria

**Keywords:** Antenatal care, Maternal health, Maternal mortality, Child health

## Abstract

The study investigates the effect of wealth on maternal health care utilization in Ghana via its effect on Antenatal care use. Antenatal care serves as the initial point of contact of expectant mothers to maternal health care providers before delivery. The study is pivoted on the introduction of the free maternal health care policy in April 2005 in Ghana with the aim of reducing the financial barrier to the use of maternal health care services, to help reduce the high rate of maternal deaths. Prior to the introduction of the policy, studies found wealth to have a positive and significant influence on the use of Antenatal care. It is thus expected that with the policy, wealth should not influence the use of maternal health care significantly. Using secondary data from the 2008 Ghana Demographic and Health survey, the results have revealed that wealth still has a significant influence on adequate use of Antenatal care. Education, age, number of living children, transportation and health insurance are other factors that were found to influence the use of Antenatal care in Ghana. There also exist considerable variations in the use of Antenatal care in the geographical regions and between the rural and urban dwellers. It is recommended that to improve the use of Antenatal care and hence maternal health care utilization, some means of support is provided especially to women within the lowest wealth quintiles, like the provision and availability of recommended medication at the health center; secondly, women should be encouraged to pursue education to at least the secondary level since this improves their use of maternal health services. Policy should also target mothers who have had the experience of child birth on the need to use adequate Antenatal care for each pregnancy, since these mothers tend to use less antenatal care for subsequent pregnancies. The regional disparities found may be due to inaccessibility and unavailability of health facilities and services in the rural areas and in some of the regions. The government and other service providers (NGOs, religious institutions and private providers) may endeavor to improve on the distribution of health facilities, human resources, good roads and necessary infrastructure among other things in order to facilitate easy access to health care providers especially for the rural dwellers.

## Introduction and background

The health of women is an important non- income indicator of poverty, hence, one means of reducing poverty in developing countries is to improve the health of women. The Ghana Poverty Reduction Strategy recognizes that improving the health of the poor is crucial for reducing poverty given that, ill health is both a consequence and cause of poverty. According to the Ghana Demographic and Health Survey (GDHS) report [1], “the health care that a mother receives during pregnancy, at the time of delivery, and soon after delivery is important for the survival and well-being of both the mother and her child”. Hence, one means of achieving an improvement in the health of women is providing maternal health services, such as antenatal care (ANC) during pregnancy. Indeed the Fifth Millennium Development goal recognizes this and therefore aims to reduce maternal deaths by three- quarters by 2015 (to achieve a maternal mortality ratio of 54 per 100,000 live births) by improving the preventive health care delivered to women during pregnancy. The maternal mortality ratio is high in Ghana. According to the Reproductive and Child Health unit of the Public health division, Ghana health service (RCH/PHD-GHS) [2] the maternal mortality ratio in Ghana is 230/100,000 live births. It is worth mentioning that ANC use alone cannot address the high rate of maternal deaths, but has to be accompanied with a healthy lifestyle by the expectant mother as directed by the health care provider. It is also an indicator of access and use of maternal health care during pregnancy, and serves as a point of contact between the provider and the expectant mother which influences the expectant mother’s use of other maternal health services such as delivery and post natal care.

The World Health Organization [3] defines antenatal care (ANC) as “care before birth”, and includes education, counseling, screening and treatment to monitor and to promote the well being of the mother and baby. The RCH/PHD-GHS annual report [2], defines ANC as “the health care and education given during pregnancy”. The Ministry of Health (MoH) report [4] states that “The objective of antenatal care is to promote and maintain the health of pregnant women. It aims to establish contact with pregnant women in order to detect and manage current health problems. During this period women and their care-givers can develop delivery plans based on their needs, resources and circumstances”. It must be stated that, it is only appropriate ANC use that would significantly help in identifying and mitigating the risk factors in pregnancy. Magadi et al [5] posits that the failure to receive appropriate ANC during pregnancy can lead to undesirable pregnancy outcomes such as maternal morbidity, low birth weight for the baby or even maternal and perinatal mortality. The MoH report [4] states that,” In order for a particular woman to derive maximum benefit from antenatal care, it is essential for her to start utilizing the service early in pregnancy and to attain a minimum number of contacts with the service”. The World Health Organization [6] recommends a minimum of four ANC visits for a pregnant woman before delivery if the woman cannot make the number of visits as directed by the physician/provider. According to Villar et al. [7], recent empirical evidence has shown that four visits suffice for uncomplicated pregnancies, and more visits are only recommended in case of complications. Many mothers in developing countries however do not receive such care, contributing to the high rate of maternal deaths due to pregnancy complications. Antenatal care (ANC) coverage is defined as the percentage of women who used ANC services provided by skilled health personnel for reasons related to pregnancy at least once during pregnancy, as a percentage of live births in a given time period usually one year. The services provided constitutes screening for health and socioeconomic conditions likely to increase the possibility of specific adverse pregnancy outcomes, providing therapeutic interventions known to be effective; and educating pregnant women about planning for safe birth, emergencies during pregnancy and how to deal with them.

ANC services are provided free of charge in all government hospitals in Ghana, (and in some accredited private, NGO and religious hospitals). Some private and religious hospitals also provide ANC services, but at a fee. ANC coverage has been around 87%, but adequate utilization (expectant mothers who are able to attend the minimum of four visits) of the service has been stagnant around 60% for the past four years according to the Ministry of Health report 2007 [4]. The Initiative for Maternal Mortality Programme Assessment (IMMPACT) [8] reported that some of the main causes of death in Ghana are likely to be inaccessible and unaffordable health care. In 1985, as part of the reforms in the financial sector of the economy, user fees were introduced in health facilities in Ghana commonly referred to as the “cash and carry system”. This policy led to a reduction in the utilization of services especially among the poor. In September 2003, the policy of exempting users from delivery fees was introduced in the four most deprived regions of the country; Central, Upper East, Upper West and the Northern regions. In April 2005, the policy was extended to the remaining six regions of the country. The aim of the policy was to reduce the financial barriers in using maternity services, (which includes ANC). It was expected that this would lead to a reduction in maternal and perinatal mortality in the country. Maternal mortality is still high even after the introduction of the policy, (IMMPACT) [8]. The policy was introduced with the view that, the cost of maternal services was serving as a hindrance to utilization. It is therefore expected that after the implementation of the free maternal health policy, wealth should have an insignificant effect on the use of ANC services in the country. Indeed Abor & Abekah-Nkrumah [9] and Overbosch et al. [10] who used data prior to the introduction of the free ANC policy found a positive and significant relationship between wealth and ANC use in Ghana. This suggests that probably improving the wealth of individuals or removing the cost barrier would help improve the use of adequate ANC in Ghana. Thus, this study seeks to fill the research gap after the introduction of the policy and to investigate if wealth still has an effect on maternal health services using ANC as a proxy, and hence find out if there are other variables that also influence the use of ANC and hence other maternal health care services in Ghana.

### Wealth and antenatal care use

Wealth signifies the economic status of the individual/family. Several studies have found wealth to influence the use of health services positively. Ortiz [11] concluded that wealthier mothers have more chance of attending a first visit and additional visits than poorer mothers in Colombia. Celik and Hotchkiss [12] showed that household wealth is positively and significantly associated with choosing health facility for delivery. Similarly, Gage [13] found household poverty and personal problems to be negatively related to the use of maternal health care. Abor & Abekah-Nkrumah [9] found that, as compared to those in the poorest households, those in the poorer households in Ghana are more likely to deliver in a health facility, with those in the middle wealth quintile being more likely to use antenatal services and deliver at a health facility. Wealth is expected to have a positive relationship with ANC since the use of the service is associated with the cost of consultation and the purchase of recommended medication alongside other indirect costs such as transportation cost. Thus, it is expected that, the higher the wealth of the woman, the more likely is she to use ANC because she may be able to afford the cost and other expenses that comes with using the service. The GHDS [1] reports, that there is a “positive relationship between professional antenatal care coverage and wealth quintile, with women in the highest wealth quintile more likely to receive care from a health professional than those in the lowest wealth quintile, although the difference is small (99 and 93 percent, respectively)”. The wealth quintile is derived from a wealth index for the household. The index has been divided into five quintiles in the data set; the lowest quintile (poorest), 2nd quintile (poorer), 3rd quintile (middle), 4th quintile (wealthier) and the 5th quintile (wealthiest). The variables that were included in the calculation of the index include: ownership of house- whether the house is owned by the household head or rented, whether there is electricity in the house or not, source of drinking water- pipe borne or well, ownership or a car, the type of cooking fuel used- firewood or others, location of kitchen- whether indoors or outside and the number of rooms in the house. Hence, with the introduction of the free maternal health policy, it was expected that wealth should have an insignificant effect on maternal health care use. This study therefore seeks to validate this prediction, and further investigate if there are other variables that also influence the use of ANC, and hence maternal health care services in Ghana.

### Other socio-economic determinants of antenatal care use

Other socioeconomic variables that do influence the use of ANC are level of education, age, residence (rural/urban), geographical location (region of residence) and number of living children. These are other potential variables that are likely to either aid the expectant mother in the use of ANC or hinder her. The level of education of the mother is a very key factor that determines the level of utilization of maternal health services. According to Grossman [14], education makes a person efficient in the use of health services and may enable the individual to choose a more health conscious behavior to improve health. Henze [15], Mekonnen & Mekonnen [16], Celik [17], Ortiz [11], Alexandre et al [18], Abor and Abekah-Nkrumah [9], Tayie and Lartey [19] and Addai [20] are some studies that have found a positive and significant association between education and maternal health care use. Overbosch et al [10], concluded in Ghana that, “Women’s attitude to antenatal care seems to be influenced by their schooling, since more years of education of a pregnant woman is associated with a choice for sufficient antenatal care”. Thus, in the campaign to raise the utilization of maternal health care services, there is the need to encourage women to pursue higher education.

Age and the number of living children of the mother may also affect her use of ANC. Empirical studies on age presents mixed evidence. For instance Chandhiok et al [21] and Henze [15], found a reduction in the proportion of women obtaining ANC services with increasing age in India and Honduras respectively; on the other hand Celik [12] and Ortiz [11] found a positive relationship between ANC and age in Turkey and Colombia respectively. Thus, the influence of age on the use of maternal health cannot be determined prior to investigation. Alongside age is the number of living children of the expectant mother which proxies a mother’s experience with ANC services. The expectant mothers’ use of ANC may be influenced by her previous experience. This may be positive ( a pleasant experience at the health center) increasing the use of the service or negative ( an unpleasant experience at the health center), thereby reducing her use of ANC. Complications experienced during earlier pregnancies also have a positive association with the use of ANC. According to Overbosch et al [10], “Pregnancy is a natural process and women with some experience might consider antenatal care less necessary”. This may be due to the fact that after encountering the service, the expectant mother may think that she knows enough to take care of subsequent pregnancies. Elo [22] and Raghupathy [23] report that a higher number of previous pregnancies are associated with less use of ANC, while Magadi, Madise and Rodrigues [5] report a negative association between a higher number of previous pregnancies and early attendance to antenatal care.

Place of residence (rural/urban) and Geographical location (region) may also affect the utilization of ANC services. According to Abor & Abekah-Nkrumah [9], urban dwellers may be relatively closer to healthcare facilities than rural dwellers in most developing countries. Overbosch et. al.,[10], reports that “currently, more than a third of the rural women have to travel more than 5km to the modern provider of ANC” in Ghana. Thus, accessibility to health care services may be much easier for the urban dwellers than the rural dwellers, thereby increasing the probability of an expectant mother in the urban center using ANC compared to her rural counterpart. Celik & Hotchkiss [12], Navaneetham & Dharmalingam [24] and Abor & Abekah-Nkrumah [9] are some studies that have concluded that differential access to health care facilities between the rural and urban centers reduced utilization of maternal health care services for the rural dwellers. Closely related to this is the distance to the health facility and transportation problems one may encounter in accessing health services. Thus, these can greatly hinder the utilization of the ANC services since they serve to discourage the expectant mother who may have to travel along bad road networks or may have to travel for long distance before being able to access a health centre for ANC. Even if the mother does it the first time, further visits may be hindered due to the struggle to get to the health facility, thereby reducing adequate utilization.

## Data and methods

### Data

The study uses data from the 2008 Ghana Demographic and Health Survey (GDHS 2008). The survey is carried out every five years (In Ghana, it was carried out in 1988, 1993, 1998, 2003, 2008). A special survey on maternal health was carried out in 2007. The sample contains women within the ages of 15-49 with a live birth in the five years preceding the survey. The questionnaire consists of questions on demographic indicators, health status, illnesses, and visits to a doctor, health behavior such as questions on smoking, drinking alcohol, physical activity, and eating habits. To measure demand for ANC the number of visits to a modern health facility namely public/government hospitals, mission hospitals and private hospitals is used. This has however been ordered according to the WHO [6] protocol of a minimum of four visits before delivery, which is seen as sufficient to ensure safe delivery, and further visits are recommended only when any complications are detected by the physician. The price of ANC is not included in the estimation equations, because ANC services are provided free of charge in all public hospitals, and some private and religious hospitals for all pregnant women in Ghana. Hence an expectant mother who decides to use a private hospital that charges for the service is revealing her preference for the services of that health provider and this may be due to other factors not covered in this study. In this study, a total of 2118 women who had given birth prior to the five years preceding the survey are investigated.

### Methods

The study employs both univariate and multivariate analysis to investigate the effect of wealth and other socioeconomic variables on ANC use in Ghana. The univariate analysis involves the use of chi square to test the significance of association between ANC use and each of the predictors in the study. In reality there are a multiplicity of factors that do influence the mother in making such a decision, thus, the study goes a step further to carry out a multivariate analysis when these variables are allowed to interplay. The multivariate model is specified as a generic latent linear probability function as:

(1) H*=X’β+u

H* is the number of antenatal care visits (which has been ordered as 0, 1, 2, 3, and 4 plus visits); X is a vector of explanatory variables, and U represents the random error term that is assumed to be normally distributed.

The model above is estimated using the ordered logistic regression model. This is an extension of the logistic regression model, allowing for more than two responses. It is estimated using maximum likelihood estimation technique. The ordered logistic model is employed in this study because the dependent variable, ANC visits has been put in an ordered discrete choice from no visit to four or more visits as proposed by the W.H.O. This is to help find out the influence of wealth and other socio economic variables which influences the woman’s decision as she progresses from no visit to the recommended number of four visits. The control variables are education, number of living children, age of the mother, and distance to health facility, problems of transportation, residence and regional dummies.

## Results

The Pearson’s chi square test is used to test whether there is a significant difference in the use ANC by the various explanatory variables. The results as presented in Table1 indicate that there is a significant relationship between each of the explanatory variables and ANC use except age and employment status. The use of ANC improves with the wealth and educational level of the woman. Utilization is also higher among women in the urban areas and expectant mothers with health insurance. The results also suggest that ANC use falls with an increasing number of living children. The table also shows that Greater Accra region recorded the maximum number of expectant mothers who made the recommended visits compared to the Ashanti and the Upper West Regions with 85.4% and 85.2% respectively. The Northern Region however recorded the lowest number of the mothers who made the recommended visits (67.5%). It must however be noted that this analysis was carried out for each of the explanatory variables without controlling for the other factors that also influence the use of ANC. In the next sub-section, a multivariate analysis is conducted to assess the impact of each explanatory variable on ANC use whilst controlling for the other factors that also influence ANC use in Ghana.

**Table 1:** Univariate investigation of antenatal care use in Ghana

**Variables**	**No ANC (%)**	**1-3 visits (%)**	**At least 4 visits (%)**	**Chi- square test**
**Educational level of the mother**				82.7***
No Education	5.5	22.8	71.7	
Primary Education	5,0	20.0	75.0	
Secondary Education	1.9	10.2	88.0	
Higher Education	0.0	0.0	100	
**Wealth Quintiles**				145.8***
Lowest	6.2	25.8	68.0	
2nd	5.6	20.4	74.0	
3rd	3.0	17.7	79.3	
4th	1.3	6.5	92.2	
Wealthiest	0.4	3.3	96.3	
**Residence**				
Urban	1.3	8.2	90.5	89.8***
Rural	5.3	21.6	73.1	
**Health Insurance**				
No	5.3	21.5	73.2	68.2***
Yes	1.8	10.4	87.8	
**Employment status**				
Not Employed	1.5	17.2	81.3	4.61
Employed	4.2	16.8	79	
**Region**				
Western Region	5.4	20.3	74.3	85.1***
Central Region	3.9	19.2	76.9	
Greater Accra Region	2.4	4.4	93.1	
Eastern Region	3.3	22.8	73.9	
Ashanti Region	1.9	12.7	85.4	
Brong Ahafo Region	2.9	16.9	80.2	
Northern Region	4.9	27.5	67.5	
Upper East Region	4	13.6	82.5	
Upper West Region	2.9	11.9	85.2	
**Age of woman**				
19 years and below	1.9	24.5	73.6	6.06
Between 20 and 35 years	3.8	16.6	79.7	
35 years and above	4.4	16.1	79.5	
**Number of Children**				
1 child	1.5	15.0	83.5	27.2 ***
2 -4 children	3.6	15.3	81.0	
5 or more children	6.1	21.0	72.9	

*** indicates there exists significant differences within that predictor in the use of ANC.

The results in Table2 suggest that wealth, education, number of living children, problems with transportation, ownership of health insurance, age of woman and residence (rural/urban) are important predictors of the use of ANC in Ghana. The results also show regional variations in the use of ANC and differential use between the rural and urban dwellers. Thus, these variables together do influence the use of ANC and other maternal health services in Ghana, and therefore any targeted intervention should focus on these characteristics to be able to achieve the desired results of reducing maternal mortality through child birth.

**Table 2:** Multivariate investigation of antenatal care use in Ghana

**Variable**	**Coefficients**	**Linearised Standard Errors**	**t stat.**	**P value**
**Wealth Quintiles (Ref: Lowest quintile)**				
2nd quintile	0.24	0.14	1.79*	0.07
3rd quintile	0.42	0.16	2.66**	0.01
4th quintile	0.90	0.17	5.38***	0.00
Wealthiest quintile	1.32	0.19	6.79***	0.00
**Educational level of the mother (Ref: No education)**				
Primary	-0.07	0.13	-0.50	0.62
Secondary plus	0.24	0.14	1.69*	0.09
**Other socio economic indicators**				
Distance is a problem to health facility	0.10	0.15	0.70	0.49
Transport is a problem to health facility	-0.31	0.15	-2.05**	0.04
Number of living children	-0.15	0.03	-4.37***	0.00
Residence (ref: Rural)	0.28	0.14	2.05**	0.04
Age of Woman in years	0.05	0.01	4.68***	0.00
Health Insurance (Ref: Insured)	0.40	0.09	4.48***	0.00
Employment Status (Ref: Not employed)	0.15	0.14	1.09	0.27
**Administrative Regions (Ref: Ashanti region)**				
Western Region	0.23	0.24	0.96	0.34
Central Region	-0.08	0.24	-0.36	0.72
Greater Accra Region	0.32	0.20	1.60	0.11
Volta Region	-0.24	0.22	-1.09	0.28
Eastern Region	-0.40	0.20	-1.95**	0.05
Brong Ahafo Region	0.18	0.21	0.82	0.41
Northern Region	-0.24	0.18	-1.30	0.19
Upper East Region	0.79	0.31	2.50**	0.01
Upper West Region	0.44	0.20	2.20**	0.03

Number of Observation = 2118 F( 22, 386) = 17.82 Prob > F = 0.000.

***indicates significant at 1% ** significant at 5%, * significant at 10%.

## Discussion of results

The paper sought to investigate the impact of wealth and other socio economic determinants of maternal health care use via ANC use in Ghana since the inception of the free maternal health policy to reduce financial barriers in the use of maternal health services. The introduction of the free maternal health policy was with the assumption that there was a significant financial barrier to the use of maternal health services, and this contributed to the high incidence of maternal mortality in the country. Hence, offering a free service will help expectant mothers in their use of maternal health services. In such a case, the effect of wealth on the use of the service should be insignificant. The results however suggest that wealth still has a positive and significant influence on the use of ANC, contrary to expectation that it should not since the service is free. Women in higher wealth quintiles are more likely to make more ANC visits than women in the lowest wealth quintile. We can infer that even though the service is provided freely, it may come with costs either directly or indirectly and those with the resources are more likely to afford it. In general, it can be concluded that, even though maternal health care services are rendered free of charge in Ghana, wealth which signifies the financial position of the individual is still a challenge in the use of these services. It still hinders the utilization rates; expectant mothers may still use the services, but not adequately as recommended by the WHO (in the case of ANC minimum of four visits before delivery) to ward off any health effects of child birth and hence to reduce the rate of maternal and child deaths through delivery. Thus, to improve the use of ANC, there is the need to go beyond providing the free services to finding means of support for expectant mothers. This can be in the form of the provision of necessary drugs and more importantly ensuring that these recommended drugs are available at the health facility to the expectant mothers. This will help reduce the cost involved in purchasing the drugs when it is not available because this can hinder the mother’s use of the services. Again, most of the expectant mothers end up spending the entire day at the health center for their check-ups. This is a form of indirect cost especially to the mothers in the informal sector who may have to go to the market to earn a daily living. Hence, there may be the need to increase the number of physicians/service providers attending to them at the health center so that they would be able to leave early. Thus, these can be done to complement the free delivery policy, and can help improve the use of adequate ANC and other maternal health services, and therefore reduce the rate of maternal deaths through child birth in Ghana.

Education, residence and transportation to the health centre have also been found to influence the use of ANC. Women with secondary or higher levels of education are more likely to use adequate ANC compared to those without education. Thus, improving the education of the mother in Ghana, will contribute greatly to the use of maternal and ANC services by women and thus help in reducing maternal and child mortality in Ghana. Specifically, women should be encouraged to pursue education beyond the primary level as the study has found that women with higher levels of education tend to make adequate use of ANC. Also, it may be possible that the less educated (especially in the rural centers) are not adequately informed about the services being offered freely and also about the adequate utilization for each pregnancy, hence it may be necessary for the National Commission for Civic Education in Ghana (NCCE) to intensify the education on the use of maternal health care services probably through the mass media (radio, television, print media) and the community announcements, especially in the rural centers where the use of mass media may not be very effective. Thus, we need to continue educating expectant mothers and women in general on a daily basis on the use of these maternal health services and to increase awareness in the communities. Expectant mothers in urban areas also tend to use more ANC compared to their counterparts in the rural areas. The positive effect might indicate that distribution of facilities between the urban and the rural areas are in favor of the urban dwellers, putting the rural dwellers at a disadvantage in terms of availability and accessibility of the services provided. Hence the Government, NGO’s and religious health care providers should make an effort to set up health centers in the rural areas to help improve utilization. This may help to also eliminate the problem of transportation if these facilities are set up in close range for the use of the rural communities. This also applies to the various regions as there exist considerable regional variations in the use of ANC.

The age of the expectant mother, number of living children and health insurance are other factors that also influence the use of ANC in Ghana. Older women are more likely to use more ANC compared to younger women. Following the predictions of the Grossman model [22]), age increases the rate of depreciation of the health of the individual, it may therefore be possible that, the older women may patronize health services more than the younger ones, and ANC services will not be an exception. There is therefore the need to educate the younger mothers on the need to use ANC and other maternal health services. ANC use also falls with increasing number of children. This may be due to the fact that once a woman has gone through the experience of child birth, she may be reluctant to undertake ANC visits for later pregnancies either due to a bad experience with the service or probably she begins to think that she has an idea of what is required of her during pregnancy. Hence, the campaign to increase the use of ANC should place a lot of focus on expectant mothers who have had the experience of child birth on the need to use adequate ANC for each pregnancy to avoid any health effects of childbirth. Expectant mothers with health insurance tend to use more ANC than those without health insurance.

## Conclusion

The study investigated the effect of wealth on maternal health care use via the use of Antenatal care (ANC) in Ghana using data from the 2008 Ghana Demographic and Health Survey (GDHS 2008). The study hypothesized that, wealth does not influence utilization of ANC services since the introduction of the free maternal health care policy which sought to remove the cost barriers to the use of ANC. Hence, expectant mothers are expected to achieve the minimum recommended number of four visits per the World Health Organization (W.H.O) protocol before delivery. Using both univariate and multivariate analysis the results have suggested that wealth significantly influences the use of ANC in Ghana. Thus, even though the free maternal health policy may have increased the utilization of ANC and maternal health care services in Ghana, adequate utilization has still not been achieved because affordability as proxied by wealth is still a problem to some expectant mothers, especially, those within the lowest wealth quintiles. It is thus recommended that to ensure adequate use of ANC and maternal health care in Ghana, some means of support is provided to women within the lowest wealth quintiles alongside the policy. This can be the provision of necessary medication prescribed for the expectant mothers, and ensuring that such drugs are at least available at the health center to relieve the mother of the cost she may have to incur in purchasing the medicine, and the availability of a number of Physicians to ensure that the mother does not spend too much time since this will serve as a discouragement to the mother in the use of the service, especially those in the informal sector, who may have to go to the market and sell to support the family as this is the case in most rural communities in Ghana. Policy should also focus on encouraging women to pursue education to at least the secondary level and the National Commission for Civic Education (NCCE) should endeavor to intensify the campaign on the use of maternal health care, especially on the free provision of the service. This may be done through the mass media (Television, radio, etc) and community announcement especially in the rural communities, since such communities are not likely to have adequate access to the mass media. There is the need to intensify education especially for the mothers who have had the experience of child birth on the need to use adequate ANC for each pregnancy. Such mothers from the study tend to use less ANC and maternal health services in general. These measures can be done alongside the policy, and hence would help in reducing the rate of maternal deaths due to child birth, and help Ghana achieve the Millennium Development Goal (Goal 5) of reducing maternal mortality.

## Competing interest

The author declares no competing interest.

## Author’s contribution

The author conceived the study, undertook the analysis and did the write up of the manuscript. The author read and approved the final manuscript.

## References

[B1] Ghana Statistical Service (GSS) Noguchi Memorial Institute for Medical Research (NMIMR), and ORC Macro: Ghana Demographic and Health Survey 2008 2009 GSS, NMIMR, and MI, Calverton, Maryland

[B2] Reproductive and Child Health Unit (Family Health Divison), Public Health Department-Ghana Health Service (RCH/PHD-GHS) Annual Report 2007 2007 Accra, Ghana

[B3] WHO The World Health Report 2000, Health systems: improving performance 2000 World Health Organisation, Geneva

[B4] Ministry of Health (MoH) Annual report 2007 2007 Accra, Ghana

[B5] Magadi MA Madise NJ Rodrigues RN Frequency and timing of antenatal care in Kenya: explaining the variations between women of different communities J Soc Sci 2000 51 551 561 10.1016/S0277-9536(99)00495-5 10868670

[B6] WHO Antenatal Care Department of Technical Working Group 1994 WHO/FRIt/msm/968 1999

[B7] Villar JH Ba’aqeel G Piaggio P Lumbiganon JM Belizán U Farnot Y Al-Mazrou G Carrolli A Pinol A Donner A Langer G Nigenda M Mugford J Fax-Rushby G Hutton P Bergsjo L Bakketeig H Berendes WHO antenatal care randomized trial for the evaluation of a new model of routine antenatal care Lancet 2001 357 1551 1564 10.1016/S0140-6736(00)04722-X 11377642

[B8] Initiative for Maternal Mortality Programme Assessment (IMMPACT) Implementation of free delivery policy in Ghana 2005 University of Aberdeen http://www.immpact-international.org

[B9] Abor and Abekah-Nkrumah The socio-economic determinants of maternal health care utilization in Ghana 2009 Submitted to African Economic Research Consortium

[B10] Overbosch Determinants of antenatal care use in Ghana,” J Afr Econ 2004 13 2 277 301

[B11] Ortiz AV Determinants of demand for antenatal care in Colombia Health Policy 2007 86 363 372 10.1016/j.healthpol.2007.12.002 18201794

[B12] Celik Y Hotchkiss DR The Socio-economic Determinants of Maternal Health Care Utilization in Turkey Soc Sci Med 2000 50 2000 1797 1806 10798333 10.1016/s0277-9536(99)00418-9

[B13] Gage AJ Barriers to the Utilisation of Maternal Health Care in Rural Mali,” Soc Sci Med 2007 10.1016/j.socscimed 17643685

[B14] Grossman M On the concept of Human Capital and the Demand for Health J Polit Econ 1972 80 223 235 10.1086/259880

[B15] Henze CE Determinants of Prenatal Care and Supplement Use: The Case of Honduras 2004 Unpublished MPH Research Project

[B16] Mekonnen Y Mekonnen A Factors Influencing the Use of Maternal Healthcare Services in Ethiopia J Health Popul Nutr 2003 21 4 374 382 15038593

[B17] Celik Y The socioeconomic determinants of alternative sources of antenatal care in Turkey Int J Health Plann Manag 2000 15 221 235 10.1002/1099-1751(200007/09)15:3<221::AID-HPM592>3.0.CO;2-A 11184655

[B18] Alexandre PK Saint-Jean G Crandall L Fevrin E Prenatal care utilization in rural areas and urban areas of Haiti Rev Panam Salud Publica 2005 18 2 84 92 16156958 10.1590/s1020-49892005000700002

[B19] Tayie FAK Laryey A Antenatal care and pregnancy outcome in Ghana, the importance of women’s education Afr J Food Agric Nutr Dev 2008 8 3 291 303

[B20] Addai I Determinants of use of maternal-child health services in rural Ghana J Biosoc Sci 2000 32 1 1 15 10676056

[B21] Chandhiok Determinants of antenatal care utilization in rural areas of India : A cross-sectional study from 28 districts J Obstet Gynecol India 2006 56 No. 1

[B22] Elo IT Utilization of maternal health-care services in Peru: the role of women’s education Health Transit Rev 1992 2 49 69 10148665

[B23] Raghupathy S Education and the use of maternal health care in Thailand Soc Sci Med 1996 43 459 471 10.1016/0277-9536(95)00411-4 8844947

[B24] Navaneetham K Dharmalingam A Utilization of maternal health care services in Southern India Soc Sci Med 2002 55 1849 1869 10.1016/S0277-9536(01)00313-6 12383469

